# Supplementation of Coenzyme Q10 among Patients with Type 2 Diabetes Mellitus

**DOI:** 10.3390/healthcare3020296

**Published:** 2015-05-21

**Authors:** Qiuhua Shen, Janet D. Pierce

**Affiliations:** School of Nursing, University of Kansas, 3901 Rainbow Blvd., Mailstop 4043, Kansas City, KS 66160, USA; E-Mail: jpierce@kumc.edu

**Keywords:** type 2 diabetes mellitus, coenzyme Q10, mitochondrial dysfunction, oxidative stress, antioxidant, adenosine triphosphate

## Abstract

Type 2 diabetes mellitus (T2DM) is a major cause of morbidity and mortality with ever increasing prevalence in the United States and worldwide. There is growing body of evidence suggesting that mitochondrial dysfunction secondary to oxidative stress plays a critical role in the pathogenesis of T2DM. Coenzyme Q10 is an important micronutrient acting on the electron transport chain of the mitochondria with two major functions: (1) synthesis of adenosine triphosphate (ATP); and (2) a potent antioxidant. Deficiency in coenzyme Q10 is often seen in patients with T2DM. Whether restoration of coenzyme Q10 will help alleviate oxidative stress, preserve mitochondrial function, and thus improve glycemic control in T2DM is unclear. This article reviews the relationships among oxidative stress, mitochondrial dysfunction, and T2DM and examines the evidence for potential use of coenzyme Q10 as a supplement for the treatment of T2DM.

## 1. Introduction

Diabetes is a major public health concern that affects 29.1 million or more than 9% of the USA population with 90%–95% cases as T2DM [1]. It was the 7th leading cause of death in the USA in 2010. Among the USA population with diabetes, 27.8% or 8.1 million are undiagnosed. According to the International Diabetes Federation Diabetes Atlas, there are 387 million people worldwide who have diabetes and it is estimated that this number will increase to 592 million by 2035 [2]. Patients with T2DM are at significantly high risk of developing serious macro- and micro-vascular complications associated with uncontrolled hyperglycemia such as heart disease, stroke, hypertension, retinopathy, nephropathy, and neuropathy. In 2012, the estimated total costs for diabetes in the USA were $245 billion with $176 billion as direct medical costs and $69 billion associated with indirect costs due to disability, work loss, and premature death [1]. Current clinical guideline for T2DM management includes healthy eating, weight control, increased physical activity, antiglycemic medications, and multifactorial risk reduction [3].

Type 2 diabetes mellitus (T2DM) is a complex and chronic disease in which the body fails to respond to the increased level of glucose due to attenuated insulin-stimulated glucose uptake with a normal amount of insulin (insulin resistance) and/or impaired insulin secretion from pancreatic β cells [3]. The pathophysiology of T2DM is complex and involves multiple factors. Insulin resistance is the underlying mechanism of developing T2DM. Secreted from pancreatic β cells, insulin plays important roles in many metabolic processes including regulation of glucose uptake, controlling membrane transport of ions to promote protein synthesis, and controlling gene transcription and cell proliferation [4]. The insulin signaling pathways involves interactions of multiple complex molecules, which are subject to various interfering factors. These include genetic abnormalities, fetal malnutrition, increased free fatty acids due to visceral adiposity, and physical inactivity. Impaired insulin action stimulates compensatory secretion of more insulin from pancreatic β cells when they are adequate to function, resulting in hyperinsulinemia. Correspondingly, hyperinsulinemia contributes to pathogenesis of metabolic syndrome, which is closely associated with T2DM. When pancreatic β cells fail to respond to insulin resistance, insulin secretion is impaired and glucose uptake is affected. Persistent hyperglycemia induces overproduction of reactive oxygen species (ROS) from the electron transport chain in the mitochondria. Although ROS is byproduct of oxidative phosphorylation in the mitochondria, excessive generation of ROS with decreased defensive mechanisms from endogenously produced antioxidants can cause oxidative stress, inducing oxidative damage to deoxyribonucleic acid (DNA), proteins, and lipids. Specifically, the mitochondria are vulnerable to oxidative stress due to the proximity to the ROS sources, leading to abnormalities in mitochondrial functions or mitochondrial dysfunction. There is accumulating evidence indicating that mitochondrial dysfunction secondary to oxidative stress contributes to the pathogenesis of T2DM and the associated complications [5].

Coenzyme Q10 (CoQ10) is an endogenously synthesized lipid-soluble micronutrient that is found in most living cells in the body. It is a key component in the electron transport chain of the mitochondria, serving as an electron transporter to transfer electrons from nicotinamide adenine dinucleotide (NADH), succinate, and glycerol-3-phosphate at complexes I and II to complex III in the process of adenosine triphosphate (ATP) synthesis. In addition, CoQ10 is a potent antioxidant that scavenges free radicals and provides protection to cells from oxidation. Studies have found that patients with T2DM often have deficiency in CoQ10 as their plasma levels of CoQ10 are significantly lower when compared to normal healthy individuals [6,7,8]. Because of its antioxidant property, it is plausible that deficiency in CoQ10 may impair the body’s defensive mechanisms against oxidative stress induced by hyperglycemia in T2DM [9,10]. It has been suggested that exogenous supplementation of CoQ10 could potentially attenuate mitochondrial dysfunction induced by oxidative stress, thus improving glycemic control in T2DM [11].

The purposes of this article are to: (1) review the relationships among oxidative stress, mitochondrial dysfunction, and T2DM; and (2) evaluate the effects of coenzyme Q10 as a supplement to the treatment of T2DM.

## 2. Mitochondria Function and Coenzyme Q10

### 2.1. Mitochondrial Function

Mitochondria are important organelles found in every cell in the body, playing roles in energy metabolism important for the survival and proliferation of cells. Often times, the mitochondria are called “the power house of the cell” [5]. This is because the majority of ATP, the source for cellular energy, is generated in the mitochondria by oxidative phosphorylation. The key mitochondrial function occurs in the electron transport chain on the inner membrane, which has many folds forming cristae. This significantly increases the surface area of the inner membrane, thus enhancing the productivity of cellular respiration. The electron transport chain consists of five complexes, including complex I, II, III, IV, and V. Complex I, also called ubiquinone NADH dehydrogenase, is responsible for oxidation of NADH and transfer electrons to ubiquinone, the oxidized form of CoQ10. Complex II, or succinate dehydrogenase, oxidizes succinate into malate and transfer electrons to ubiquinone. At complexes I and II, ubiquinone receives electrons and is reduced to ubiquinol, which carries the electrons to complex III (ubiquinol-cytochrome c reductase). During this process, two protons are generated. Complex IV, or cytochrome c oxidase, plays roles in transferring electrons from cytochrome c to oxygen, producing water and another two protons. When the protons are pumped out of the matrix into the intermembrane space, an electrochemical gradient is created. At complex V (ATP synthase), the transmembrane potential created by proton gradient is used to facilitate the synthesis of ATP from the phosphorylation of ADP [12].

### 2.2. Reactive Oxygen Species

Reactive oxygen species (ROS) are highly reactive molecules that contain oxygen and unpaired electrons. Under normal condition, ROS are a natural byproduct of oxidative phosphorylation in the mitochondria. Superoxide and hydroxyl radicals are examples of ROS. Superoxide is generated when electrons are added to oxygen during the electron transport. It can be converted to hydrogen peroxide by superoxide dismutase. Hydrogen peroxide can be further reduced to hydroxyl radicals [12]. ROS can cause oxidative damage to DNA, lipids, and proteins due to their highly reactive properties. There are endogenous defensive mechanisms such as antioxidants in the body to counterbalance ROS that are naturally produced during oxidative phosphorylation. However, when there is excessive production of ROS under various pathophysiologic disease processes and/or significantly impaired antioxidant defensive mechanisms, oxidative stress occurs. It has been known that oxidative stress contributes to an increased rate in mutation of mitochondrial DNA, leading to mitochondrial dysfunction [13].

### 2.3. Coenzyme Q10

Coenzyme Q10 is a lipid-soluble micronutrient that is endogenously synthesized in the body. It is a key component in the electron transport chain in the mitochondria. The two major functions of CoQ10 are to: (1) promote synthesis of ATP; and (2) serve as a potent antioxidant. In its first function, CoQ10 facilitates oxidative phosphorylation process by transferring electrons along the electron transport chain between complexes I, II, and III. In its second function, CoQ10 is one of the most active scavengers for ROS and provides protection to mitochondrial membrane proteins, lipids, and DNA from oxidative damage. There are two forms of CoQ10: Ubiquinone is the fully oxidized state of CoQ10 and ubiquinol is the fully reduced form of CoQ10. Ubiquinol is the active form of CoQ10 and serves as a potent antioxidant since it holds electrons loosely, and can easily give up one or two electrons to neutralize ROS. Coenzyme Q10 can also help regenerate other antioxidants, such as vitamin E [14].

Recent studies discovered additional roles of CoQ10 in the mitochondria. For example, CoQ10 is found to not only facilitate electron transport in ATP production, but also contribute to stabilization of mitochondrial permeability transition pore and protection against apoptosis and autophagy [15]. A study by Yubero-Serrano *et al.* [16] reported that supplementation of CoQ10 in Mediterranean diet was effective in modifying gene expression related to endoplasmic reticulum stress. In addition, there is a close relationship between CoQ10 synthesis and mitofusin-2, which is an essential mitochondrial membrane protein involving mitochondrial fusion [17]. It offers protection to mitochondrial mass by preserving mitochondrial cristae structure and increasing the number and volume density of mitochondria, reported in a study by Noh *et al.* [18].

### 2.4. Role of Mitochondria in Pancreatic Beta Cells

Homeostasis of glucose and insulin secretion is important to maintain optimal glucose level. This greatly depends on the ATP generated by the mitochondria in the β cells from the metabolism of glucose. In brief, insulin secretion from β cells includes the following steps [5]. First, glucose is transported into β cells via glucose transporters-1, where it is converted to pyruvate through glycolysis. Second, pyruvate is transferred into the mitochondria and broken down by the tricarboxylic acid cycle. Adenosine triphosphate (ATP) is generated when electrons are transferred by the electron transport chain and proton gradient is created. Third, ATP/ADP ratio increases when more ATP is produced, resulting in closure of ATP-sensitive K^+^ channels and depolarization of voltage-sensitive Ca^2+^ channels. Fourth, influx of Ca^2+^ triggers exocytosis of insulin secretory vesicles [5].

## 3. Mitochondrial Dysfunction, Oxidative Stress and Diabetes

**Figure 1:**
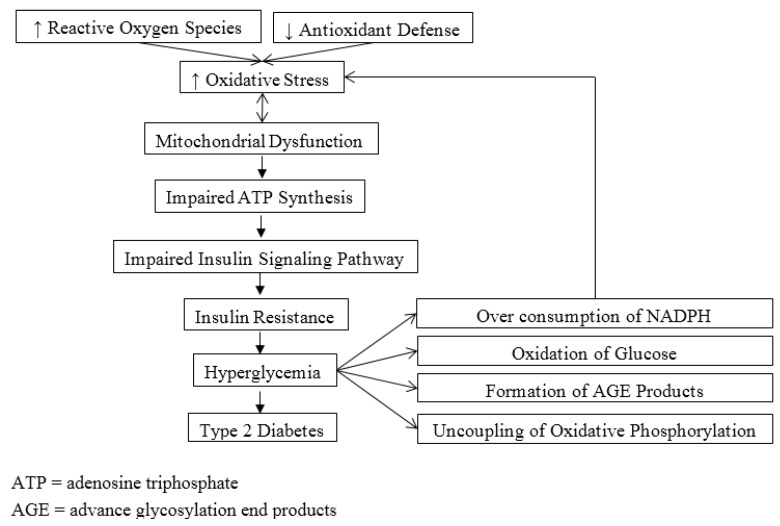
Scheme of the roles of oxidative stress in pathogenesis of type 2 diabetes.

Mitochondrial dysfunction is found to closely associate with obesity and T2DM. In obesity, there is increased supply of nutrient substrates to the mitochondria due to increased free fatty acid levels. This can cause significant increase production of ROS and intracellular fat accumulation. Both can lead to mitochondrial dysfunction by compromising the ability to oxidize fat, resulting in interference with insulin signaling pathway and worsening the state of insulin resistance [19,20]. This eventually contributes to the pathogenesis of T2DM. Another primary cause for increased oxidative stress and mitochondrial dysfunction in T2DM is hyperglycemia. The four key mechanisms associated with hyperglycemia-induced oxidative stress include over consumption of NADPH via activation of the polyol pathway, oxidation of glucose, formation of advanced glycosylation end-products, and uncoupling of oxidative phosphorylation and endothelial nitric oxide synthase [21]. There are two downsides of uncoupling of oxidative phosphorylation. First, the generation of ATP is greatly impaired when electron transport is inhibited at complex III due to increased NADH and glycerol-3-phosphate, leading to bioenergetics deficiency. Second, increased electrons from NADH and glycerol-3-phosphate are then transferred to oxygen, resulting in significant generation of superoxide. Figure 1 illustrates the roles of oxidative stress in the pathogenesis of T2DM. Mitochondrial dysfunction also plays a role in pathogenesis of diabetic micro- and macro-vascular complications associated with abnormalities in endothelial function. Impaired production of nitric oxide by endothelial cells under the condition of oxidative stress contributes to ineffective relaxation of smooth muscle cells. Alterations in mitochondrial function associated with increased production of ROS and excessive consumption of antioxidants have been attributed as significant factors for complications of T2DM, especially nephropathy [9]. This was supported by studies that showed elevated generation in mitochondrial superoxide and hydrogen peroxide was associated with development and progression of diabetic nephropathy [22,23,24].

There is convincing evidence that mitochondrial dysfunction secondary to oxidative stress plays a significant role in the pathogenesis of T2DM and its complications [25,26]. This has stimulated increased interest in using CoQ10 as a nutrient supplement due to its antioxidant property along with antidiabetic treatments in T2DM.

## 4. Coenzyme Q10 Deficiency in Type 2 Diabetes

Deficiency in CoQ10, particularly ubiquinol (the reduced form of CoQ10) is often observed among patients with T2DM. The ratio of ubiquinol/ubiquinone is used as a marker to detect oxidative stress [27]. Increased level of ubiquinone (oxidized form) associated with decreased level of ubiquinol (reduced form) indicates ineffective conversion between ubiquinone and ubiquinol and reduced capacity to counteract free radicals. It was found that the conversion of ubiquinone to ubiquinol is often impaired in many diseases [28]. For example, Ates and associates [6] found that the ratio of ubiquinol/ubiquinone was significantly lower among T2DM patients with retinopathy than normal healthy subjects (0.26 ± 0.16 *vs.* 1.41 ± 0.68, respectively). Correspondingly, the plasma level of malondialdehyde (MDA), a marker for lipid peroxidation [29], was significantly higher among T2DM patients. There was a significant negative correlation between ubiquinol/ubiquinone ratio and plasma level of MDA [6]. El-ghoroury *et al.* [7] reported very similar findings that the level of CoQ10 was significantly lower and the level of MDA was significantly higher in patients with T2DM than normal healthy individuals. A significantly negative correlation between CoQ10 and HbA1c was also found. Furthermore, the ubiquinone/ubiquinol ratio was consistently higher following breakfast and throughout the day among patients with T2DM than healthy individuals, suggesting increased oxidative stress from postprandial hyperglycemia [8]. When stratified by fasting glucose statuses, ubiquinone/ubiquinol ratio showed a stepwise significant elevation from normal glucose tolerance (NGT), impaired fasting glucose (IFG), and T2DM [26]. More specifically, 90% of the total CoQ10 was the reduced form, ubiquinol, among individuals with NGT; while it was only 24% in patients with T2DM. It was proposed that CoQ10 plays an important role in the pathogenesis of T2DM [30]. Furthermore, it was first suggested by Sourris *et al.* [9] that deficiency in CoQ10, particularly ubiquinone, is a precipitating factor for diabetic nephropathy. This was based on the evidence showing that there was significant lower level of ubiquinone in the renal cortex and mitochondria from db/db mice which is prone to develop diabetic nephropathy. Coenzyme Q10 deficiency in T2DM is most likely attributed to depletion in response to excessive oxidative stress. It is worth noted that plasma CoQ10 levels were used as the surrogate for tissue CoQ10 in all of the above clinical studies. Although it is needed to establish the relationship between plasma and tissue CoQ10 levels, difficulty in obtaining tissue samples in these clinical studies is the primary issue [31]. In addition, many of the studies did not clearly discuss the methods used to prevent the oxidation of ubiquinol when extracting CoQ10 from plasma sample. Caution is needed when interpreting these results.

## 5. Supplement Use of Coenzyme Q10 in Type 2 Diabetes

The mitochondrial FAD-dependent glycerol-3-phosphate dehydrogenase (G3PD) is the rate-limiting enzyme that locates in the mitochondrial inner membrane. It plays roles in catalyzing the transfer of electrons from glycerol-3-phosphate to CoQ10. The activity of G3PD has physiologic impact on the function of β cells. The activity of G3PD was impaired in pancreatic islets in rodent models of type 2 diabetes or cadaveric human islets from patients with type 2 diabetes [32,33,34,35]. There was speculation that increased concentration of CoQ10 in the mitochondrial inner membrane could optimize the impaired G3PD activity in pancreatic islets [36]. This was based on the observation that supplementation of exogenous CoQ10 to patients with T2DM increased more than 31% of the activity of succinate dehydrogenase [37]. The mitochondrial succinate dehydrogenase is also a flavoprotein in the mitochondrial inner membrane that donates electrons to CoQ10. It was further speculated that an optimal level of CoQ10 could also benefit activity of the Krebs cycle which is dependent on NADH dehydrogenase and succinate dehydrogenase [36].

Table 1 presents a summary of the studies that examined the effects of supplemental CoQ10 on the glycemic control among T2DM. It was first investigated in two studies [38,39]. The study by Shigeta *et al.* [38] showed that supplementation of 120 mg CoQ7 reduced glucose level by ≥20% in 67% of the 39 patients with diabetes. According to Werbach [40], daily administration of 60 mg CoQ10 to 15 patients with diabetes increased insulin synthesis and secretion and improved glycemic control [39]. Although positive effects of CoQ10 on glycemic control and insulin synthesis and secretion by pancreatic β cells were reported, none of these three studies included placebo control groups.

**Table 1:** Effects of Coenzyme Q10 Supplementation on Glycemic Control among Patients with Type 2 Diabetes.

Authors and Year of Publication	Study Design and Subjects	Form and Dosage of Coenzyme Q10	Results
Shigeta *et al.* 1966 [38]	39 patients with diabetes	CoQ7 120 mg for 2–18 weeks	There was significant decrease in glucose level (≥20%) in 67% of patients
Shimura *et al.* 1981 [39]	15 patients with diabetes	CoQ10 60 mg daily for 12 weeks	There was significantly increased in insulin synthesis and secretion. Glycemic control was improved
Conget *et al.* 1996 [41]	Rat islets	2, 4, and 8 µM CoQ10	There was no significant increase in release of insulin.25% increase in insulin secretion was observed when a higher concentration (8 µM) was used
Eriksson *et al.* 1999 [30]	Double-blind placebo-controlled study 23 patients with T2DM	CoQ10 100 mg twice a day for 6 months	There was no significant improvement in blood glucose and HbA_1c_ levels
Henriksen *et al.* 1999 [42]	Randomized, double-blind, placebo-controlled study 34 patients with T1DM	CoQ10 100 mg daily for 12 weeks	There was not significant decrease in HbA_1c_ and blood glucose levels
Singh *et al.* 1999 [43]	Patients with hypertension and coronary artery disease	CoQ10 60 mg twice a day for 8 weeks	There was significantly decrease in blood pressures. Fasting and 2 h plasma glucose and insulin levels were significantly reduced. There was significant increase in other antioxidants such as vitamin A, E, and C and beta-carotene. Markers for oxidative stress (TBARS, malondialdehyde, and diene conjugates) were significantly reduced.
Hodgson *et al.* 2002 [44]	74 patients with uncomplicated T2DM and dyslipidemia	CoQ10 200 mg/day for 12 weeks	HbA_1c_ level was significantly decreased. No significant improvement in fasting blood glucose and insulin. No significant change in oxidative stress status measured by F2-isoprostane
Lim *et al.* 2008 [45]	80 patients with T2DM	CoQ10 200 mg/day for 12 weeks	There was significant increase in plasma total CoQ10 level but no change in ubiquinol level. No significant improvement in HbA_1c_ level. No significant improvement in microcirculatory endothelial function.
Sena *et al.* 2008 [46]	Type 2 diabetic GK rat model	CoQ10 20 mg/kg body weight and/or α-tocopherol for 8 weeks	There was significant decrease in HbA_1c_ level. No significant improvement in fasting and 2 h blood glucose levels.
Mezawa *et al.* 2012 [10]	9 patients with T2DM 5 healthy volunteers	Ubiquinol 200 mg daily for 12 weeks (T2DM patients) Ubiquinol 200 mg daily for 4 weeks	There was significant improvement in HbA_1c_ (from 7.1 ± 0.4 to 6.8% ± 0.4%) among patients with T2DM. There was significant increase in insulin genic index (0.65 ± 0.29 to 1.23 ± 0.56) and decrease in proinsulin to insulin ratio (3.4 ± 1.8 to 2.1 ± 0.6) in healthy volunteers, indicating increased insulin secretion.
Sourris *et al.* 2012 [9]	Diabtic nephropathy rodent model	CoQ10 10 mg/kg/day for 10 weeks	There were significantly decreases in urinary albumin excretion in 24 h, albumin/creatinine ratio, and tubulointerstitial collagen deposition.
Kolahdouz Mohammadi *et al.* 2013 [47]	Randomized double-blind placebo-controlled trial 64 patients with T2DM	CoQ10 200 mg daily for 12 weeks	There was significant decrease in HbA_1c_ in the CoQ10 group.

CoQ10: Coenzyme Q10, HbA_1c_: Glycated hemoglobin, T2DM: Type 2 diabetes mellitus, TBARS: Thiobarbituric acid reactive substance.

In more recent years, researchers have explored the effects of CoQ10 on glycemic control among patients with T2DM in controlled intervention studies. Hodgson and colleagues [44] reported that daily supplementation of CoQ10 (200 mg/day) for 12 weeks significantly decreased HbA_1c_ level among 74 patients with uncomplicated T2DM and dyslipidemia. However, no significant improvement in fasting blood glucose or insulin levels was found in this study. In addition, there was no significant change in oxidative stress status measured by F2-isoprostanes. Despite that, the authors concluded that the effects of CoQ10 on oxidative stress could not be ruled out because F2-isoprostanes assess oxidative stress in a more systemic way and CoQ10 may play roles at the cellular or sub-cellular level. Similar findings were reported in a study by Sena *et al.* [46] that tested the effects of CoQ10 (20 mg/kg body weight) and α-tocopherol in a diabetic GK rat model. In contrast, Singh and associates [43] found that use of CoQ10 (60 mg twice daily) for 8 weeks by patients with hypertension and coronary artery disease was effective in reducing blood pressures and fasting and 2 h plasma insulin and glucose levels. In addition, significant increase in antioxidant vitamins such as vitamin A, E, and C and beta-carotene were observed. The levels of thiobarbituric acid reactive substance, malondialdehyde, and conjugated diene, markers for oxidative stress, were significantly reduced in the CoQ10 group. The authors proposed that CoQ10 provides protection to β cells through its antioxidant property and down-regulation of insulin receptors to improve insulin action. In addition to its efficacy in improving glycemic control and insulin secretion, CoQ10 was also found to provide protection to the kidneys in the event of diabetic nephropathy [9]. The study showed that the amount of urinary albumin excretion in 24 h, albumin/creatinine ratio, and tubulointerstitial collagen deposition were significantly attenuated by CoQ10 (10 mg/kg/day for 10 weeks) in a diabetic nephropathy mice model. Using a rodent model, Yokoyama *et al.* [48] demonstrated there was a 12% improvement in coronary vasodilation induced by bradykinin after cardiac ischemic perfusion among rats pre-treated with CoQ10. The positive effects of CoQ10 on glycemic control among patients with T2DM were further demonstrated in the most recent studies by Mezawa *et al.* [10] and Kolahdouz Mohammadi *et al.* [47]. This was the first time that ubiquinol (200 mg daily), the reduced form of CoQ10, was examined among a small sample size of patients with T2DM (*n* = 9) for 12 weeks [10].

In contrast, there were several studies reporting conflicting findings. For example, Conget and colleagues [41] investigated the effect of various concentrations (0, 2, 4, and 8 µM) of CoQ10 on β cell function *in vitro* using rat islets and they found that CoQ10 did not produce significantly increased release of insulin. However, a 25% increase in insulin secretion was observed when a higher concentration (8 µM) was used. In a double-blind placebo-controlled study conducted by Eriksson *et al.* [30], 23 patients with T2DM were randomly assigned to receive oral CoQ10 (100 mg, twice a day) or placebo for 6 months. No significant improvement in glycemic control (*i.e.*, blood glucose level, HbA_1c_) was observed in the CoQ10 group compared to the control group. The findings were similar to those reported by Henriksen *et al.* [42] who tested the use of CoQ10 (100 mg daily for 12 weeks) by patients with T1DM. The sample size for the study by Eriksson *et al.* [30] was small and the study might be underpowered. The most recent study by Lim and associates [45] also showed that there was no significant change in HbA_1c_ level among 80 patients with T2DM who took 200 mg CoQ10 daily for 12 weeks.

Most of these studies were conducted in the 1990s when ubiquinol, the reduced form of CoQ10, was not available in the market as a supplement and ubiquinone, the oxidized form of CoQ10, was used instead. Many patients with T2DM are usually older than 40 years when the ability of the body to convert ubiquinone to ubiquinol starts to deteriorate during aging. In addition, the efficiency of the conversion is significantly impaired in advanced disease process such as T2DM [28]. This was further supported by the study by Lim *et al.* [45], which showed that oral CoQ10 supplement (ubiquinone, 200 mg daily for 12 weeks) increased the level of total CoQ10 by three times, but failed to increase the ratio of ubiquinol. In contrast to ubiquinone, ubiquinol is the key to counteract with ROS, acting as the antioxidant. Therefore, the findings of no significant improvement in glycemic control, or insulin secretion by supplementation of ubiquinone in T2DM may be attributed to the impairment of ubiquinone to be converted to ubiquinol. Dosing of supplementation of CoQ10 could be another factor that may account for the inconsistent results reported by previous studies. A low dosage of CoQ10 (*i.e.*, 100 mg daily) most likely would not help reach the optimal therapeutic level, especially when deficiency of CoQ10 is present. Further research that examines the effectiveness of ubiquinol in T2DM is warranted.

In addition to CoQ10, researchers also investigated the effects of other antioxidants (e.g., anthocyanin, vitamin C and E, and resveratrol *etc*.) in the treatment of T2DM. The results were also mixed. For example, Li and associates [49] found that daily supplementation of purified anthocyanin for 24 weeks was effective to lower fasting plasma glucose and insulin resistance, and decrease dyslipidemia (LDL cholesterol, triglycerides, apolipoprotein B, and apolipoprotein C) among 58 patients with T2DM. In a randomized double-blinded study among patients with T2DM, supplementation of vitamins C and E for 3 months significantly decreased the levels of fasting blood glucose and HbA_1c_ compared to the placebo group [50]. Bhatt and colleagues [51] reported that 3-month supplementation of resveratrol among patients with T2DM significantly improved HbA1c levels in a randomized, controlled clinical trial. Similar results were observed in the study by Movahed *et al.* [52]. Furthermore, antioxidants were found to restore endoplasmic reticulum function and preserve β cell function in a T2DM mice model [53]. However, there were studies that reported only marginal or no significant clinical benefits of antioxidants as an adjunct therapy for T2DM [54,55,56,57]. Once again, factors such as design, sample size, and dosing of these studies could have impacted the interpretations of the findings.

## 6. Conclusions

It is clear that mitochondrial dysfunction secondary to oxidative stress contributes to the pathogenesis of T2DM. Deficiency in CoQ10 is often present among patients with T2DM due to various reasons. As a potent antioxidant, CoQ10 is assumed to scavenge excessive ROS and provide protection to cells, especially mitochondria from oxidative damage. Therefore, restoration of CoQ10 level among patients with T2DM by supplementation of exogenous CoQ10 could potentially alleviate oxidative stress, preserve mitochondrial function, and eventually lead to improvement of glycemic control. This hypothesis was partially supported by several studies [9,10,38,39,43,44,46,47,48]. However, there were also studies that report no significant improvement in T2DM [30,41,42,45]. Thus, it is still unclear, or impossible to make a definitive conclusion on whether supplementation of CoQ10 would provide beneficial effects for patients with T2DM with current available evidence. Large randomized clinical trials are needed to further investigate its effects in T2DM, using ubiquinol (the reduced form of CoQ10) with higher dosage.
